# The Prevalence of Limited Health Literacy and Its Associated Factors among Elderly Patients Attending an Urban Academic Primary Care Clinic in Malaysia

**DOI:** 10.3390/ijerph18179044

**Published:** 2021-08-27

**Authors:** Siti Nur Hidayah Abd-Rahim, Mohamed-Syarif Mohamed-Yassin, Suraya Abdul-Razak, Mohamad Rodi Isa, Noorhida Baharudin

**Affiliations:** 1 Department of Primary Care Medicine, Faculty of Medicine, Universiti Teknologi MARA, Jalan Prima Selayang 7, Batu Caves 68100, Malaysia; adrenalina4c@yahoo.com (S.N.H.A.-R.); suraya617@uitm.edu.my (S.A.-R.); noorhida8229@uitm.edu.my (N.B.); 2 Institute of Pathology, Laboratory and Forensic Medicine (I-PPerForM), Universiti Teknologi MARA, Sungai Buloh 47000, Malaysia; 3 Cardio Vascular and Lungs Research Institute (CaVaLRI), Pusat Perubatan UiTM, Kampus Sungai Buloh, Sungai Buloh 47000, Malaysia; 4 Department of Public Health Medicine, Faculty of Medicine, Universiti Teknologi MARA, Jalan Hospital, Sungai Buloh 47000, Malaysia; rodi@uitm.edu.my

**Keywords:** health literacy, elderly, older adult, aging, primary care, Malaysia

## Abstract

Limited health literacy (HL) is linked to many negative health outcomes, including poor self-management of chronic diseases and medication adherence among patients. There are a lack of data regarding HL in the elderly population in Malaysia. This study aimed to determine the prevalence of limited HL levels and its associated factors among elderly patients in an urban academic primary care clinic in Selangor, Malaysia. A cross-sectional study was conducted among 413 elderly patients (≥60 years old) who attended this academic primary care clinic between January 2020 and January 2021. Sociodemographic data, clinical characteristics, and health literacy scores were collected. Descriptive statistics (median with interquartile ranges (IQR), frequency, and percentages) and multiple logistic regression were utilized. The prevalence of limited HL in our population was 19.1% (95% CI: 15.3, 23). The middle-old (70–79 years) and very-old (≥80 years) age groups were more likely to have limited HL (aOR 4.05; 95% CI: 2.19, 7.52 and aOR 4.36; 95% CI: 1.02, 18.63, respectively). Those with at least secondary school education (aOR 0.06; 95% CI: 0.02, 0.24) and those who found medical information via the internet/television (aOR 0.21; 95% CI: 0.05, 0.93) had lower odds of having limited HL. In conclusion, having limited HL levels was not common among elderly patients in this primary care clinic. Further studies involving rural and larger primary care clinics in Malaysia are required to support these findings.

## 1. Introduction

Health literacy (HL) has been identified as a critical component to improve health and well-being while reducing health disparities [1]. The World Health Organization (WHO) defines HL as the “cognitive and social skills which determine the motivation and ability of individuals to gain access, understand and use information, which promotes and maintain good health” [1]. The purpose of HL is to help people make health-related decisions and take appropriate actions to manage their health [1,2]. The ability to find, understand, appraise, and apply information related to health is essential in order to prevent disease and promote good health [1,2].

Limited HL is linked to increased healthcare problems, including medical and medication mistakes, poor adherence to prescribed medications, difficulty communicating with healthcare providers, and a lack of understanding of disease procedures and self-management abilities in people with chronic diseases such as diabetes, hypertension, and heart disease [3,4,5]. Limited HL has also been found to weaken elderly people’s autonomy and limit their independence [4,6]. Hence, empowering patients to make meaningful health decisions and participate in health-promoting and sustaining activities are critical for improving their health outcomes [7]. Better health outcomes and lower utilization of healthcare services are the outcomes of good HL levels in terms of cost efficiency, frequency, and length of hospitalization [8,9,10].

The elderly population makes up 30% of the global population [11]. Most East Asian middle-income countries are aging at a rapid pace, faster than Europe and the United States did last century [12]. Over the last half-century, Malaysia’s demographic age structure has changed significantly [13]. Malaysia follows the definition of the United Nations World Assembly on Ageing in Vienna in 1982 to define the elderly as those aged 60 and above [14]. Like the rest of the world, it is experiencing an increase in the number of elderly people [13,15]. This is attributable to a variety of causes, including lower fertility rates, lower infant mortality, and improved healthcare facilities [9,13,16]. As a result of this, the overall population of elderly individuals increased from 7.9% in 2010 to 10.7% in 2020 [15]. Therefore, Malaysia is forecasted to become an aging country by 2030, with the percentage of old people rising to about 20% [14]. This will have a substantial impact on the country’s social, cultural, and economic landscapes, as well as its healthcare delivery system [9,10,12,14,17].

Hence, Malaysia must plan to embrace this issue within a shorter time frame compared with other developed countries such as Japan, France, and Germany, who have had decades or even centuries to prepare for the impacts of their ageing population [14]. Consequently, this population group has been designated as one of Malaysia’s national health priority groups because they are more likely to develop age-related diseases, have higher morbidity, use health services more frequently, are more likely to develop complications and have a higher demand for specialist services [14,18]. They are the biggest group of patients who utilize health care services in Malaysia, accounting for almost 40% of overall healthcare resources [9,19,20,21]. Furthermore, they are more likely to be admitted to the hospital and have longer periods of hospitalization [9,21]. The elderly are also the most common category of patients that utilize primary care services [19,20].

The elderly is the most prevalent group with limited HL in many countries such as the USA, UK, Germany, Brazil, China, Taiwan, and Vietnam [8,22,23,24,25,26]. The prevalence of limited HL in elderly populations worldwide ranged from 27 to 91.5% [27,28,29,30]. While most of these studies were conducted in a community setting [29,30,31,32], one was conducted among patients in a district hospital [28] and another was based on a population registry in Germany [27].

Previous studies have reported several factors that were associated with HL levels among the elderly population. These include education level, ethnicity, income, age, cognitive status, presence of chronic diseases, information seeking through television or internet, and physical performance [4,23,27,28,29,30,32,33,34,35]. Studies in China, Israel, and Germany reported that elderly persons with no or a low education had lower HL levels [27,28,29,30,33,34]. A Chinese study found that being in an ethnic minority group was associated with having limited HL [29]. Income deprivation was also associated with limited HL according to studies conducted in China and Germany [27,29,30,32]. Several studies also concluded that older age was linked to limited HL [23,28,32]. In contrast, the elderly in Finland and China with a lesser number of chronic diseases had lower levels of limited HL [4,29]. Few studies found that the elderly who sought information through television or the internet were associated with higher HL levels [23,29,35]. Finally, Eronen et al. reported that improved cognitive status was associated with higher HL levels among elderly Finns [4].

There are a lack of published data on HL among the elderly in Malaysia [36]. A recent community study among a group of elderly in an urban neighborhood setting in Shah Alam, Selangor, found that 62.6% of the elderly population had limited HL levels [31]. However, there is no published study on the prevalence of limited HL and its associated factors among elderly patients in a primary care setting in Malaysia. Hence, this study aimed to determine the prevalence of limited HL levels and its associated factors among elderly patients in an urban academic primary care clinic in Selangor, Malaysia.

## 2. Materials and Methods

### 2.1. Study Design and Population

This was a cross-sectional study conducted from January 2020 to January 2021 among elderly patients who attended an urban academic primary care clinic in Malaysia. This primary care clinic is located in the Gombak district, Selangor. Services provided here include walk-in clinics for acute presentations and health screening and appointment-based clinics for chronic disease follow up. The clinic has access to radiology, laboratory, and referral services for other specialties. An average of 80 patients attend this clinic per day. All of the doctors working there are family medicine specialists or postgraduate doctors pursuing a Master’s degree in family medicine. The vast majority of the patients reside in the surrounding areas of Selangor and Kuala Lumpur.

The patients were chosen based on the inclusion and exclusion criteria. Elderly patients aged at least 60 years who understood English or the Malay language were included. Meanwhile, the elderly who presented with acute medical conditions such as hypertensive emergency, acute coronary syndrome, or psychiatric emergencies, i.e., acute psychosis or delirium, were excluded.

### 2.2. Sample Size Determination

The sample size was determined using the “Sample size for a Proportion of Descriptive Study” formula based on the main objective of the study. The required sample size was based on the prevalence of limited HL among the elderly in Germany (66.3%) by Vogt et al. [32]. The confidence interval was taken as 95%. Based on these assumptions, the minimum required sample needed for this study was 344 patients. Taking into account a 20% non-response and non-eligible rate, this study aimed to approach at least 413 participants.

### 2.3. Study Tool

The study tool consisted of two parts. The first part was the sociodemographic and clinical characteristics. The Elderly Cognitive Assessment Questionnaire (ECAQ) is a measure for assessing cognitive impairment generated from the Mini-Mental State Examination and the Geriatric Mental Status Schedule [37]. ECAQ was developed and validated in Singapore by Kua et al. in 1992, and it shows a sensitivity of 85.3 and specificity of 91.5% with a positive predictive value of 82.8% [37]. It is available in three languages: English, Malay, and Chinese [21]. It consists of three sections: memory (three items), orientation (six items), and memory recall (one item). ECAQ is scored as the following: (i) ≥7 for normal, (ii) 5–6 for borderline dementia, and (iii) ≤4 for probable dementia.

The second part was the Health Literacy Survey Questionnaire Short Form 12 questions (HLS-SF12) version. The original HLS-SF12 version and the Malay version were developed and validated in a six Asian country study by Duong et al. [38]. It has good reliability (Cronbach’s α of 0.85) [38]. As this short questionnaire was developed based on the longer HLS-EU-Q47 questionnaire, a confirmatory factor analysis (CFA) was used to establish construct validity for the three domains of health care (HC), disease prevention (DP), and health promotion (HP) [38,39]. In the CFA, the Comparative Fit Index (CFI) and the Root Mean Square Error of Approximation (RMSEA) were used as the model fit indices. The CFI value was 0.95, which indicated an acceptable model fit. The RMSEA value of 0.06 represented a good fit. The three-domain model showed a goodness of fit with χ^2^/df (relative chi-square) of 2.38, AGFI (adjusted goodness-of-fit index) of 0.94, GFI (goodness-of-fit index) of 0.96, IFI (incremental fit index) of 0.95, and NFI (normal fit index) of 0.92 [38]. The overall results supported the fitness of the four-factor structure within each of the three domains of the HLS-EU-Q47 [38].

Each item of the HLS-SF12 assesses the perceived difficulties in finding, understanding, judging, and applying health information. It uses a four-point Likert scale with responses of 1 = very difficult, 2 = fairly difficult, 3 = fairly easy, and 4 = very easy. The HL score is calculated using the following formula: HL score = (M − 1) × (50/3), where M = mean, 1 = the minimal possible value of the mean, 3 = the range of the mean, and 50 = the chosen maximum value of the desired scale. HL scores ≤ 33 were classified as limited HL, while >33 were classified as adequate HL [38].

### 2.4. Patient Recruitment, Sampling Method, and Data Collection

Elderly patients who attended an urban academic primary care clinic in Malaysia between January 2020 and January 2021 were recruited. Every alternate elderly patient who registered at the clinic was approached in the assessment room and was invited to partake in the study. Next, they were screened for eligibility according to the inclusion and exclusion criteria. Those who were interested were given a participant information sheet containing information about the study background, purpose, and benefits. Eligible participants who consented were assured of their confidentiality, briefed about the study, and were then given the study questionnaire. Data were collected by a trained research assistant to guarantee a uniform data gathering procedure.

**Figure 1:**
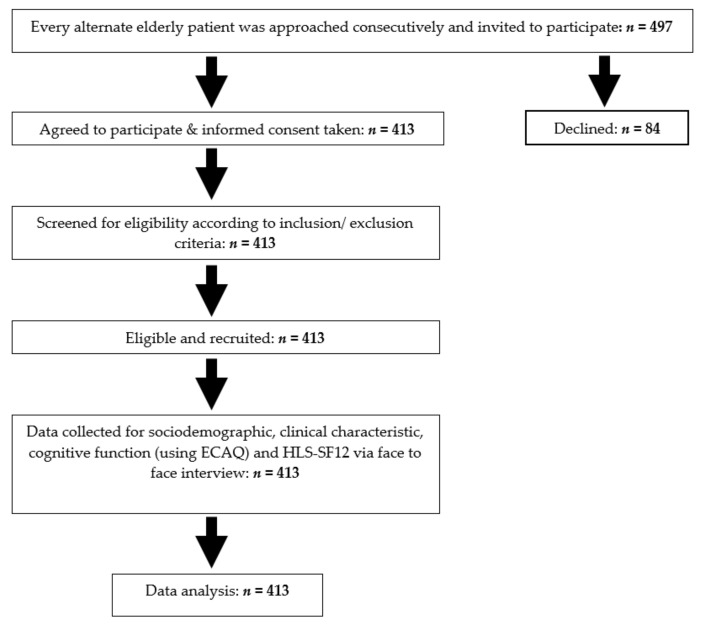
Flow chart of the study.

Sociodemographic and clinical characteristics collected included the participants’ age, gender, marital status, ethnicity, education level, occupation, household income, number of children, living arrangement, ECAQ score, smoking status, alcohol consumption, presence and number of medical illness, family history of medical illness, family members with medical training, perceived health status, accompanied by family to the clinic, finding medical information on the television or internet, and limitations on activities. HL score was determined using the HLS-SF12 questionnaire. The flowchart of this study is shown in Figure 1.

### 2.5. Definition of Terms

Regarding the sociodemographic variables, the elderly were divided into three age groups. These are the young-old (60–69 years old), middle-old (70–79 years old), and very-old (over 80 years old) [16,32,40]. Education levels were classified according to the Malaysian education system, as follows: no formal education, primary school (standard 1–6; ages 7–12), secondary school (form 1–5; ages 13–17), and tertiary education (college or university). Household incomes were categorized into two groups: low (B40) with a monthly household income of RM < 4850, and middle (M40) and high (T20) with a monthly household income of RM ≥ 4850 [41].

### 2.6. Data Entry and Statistical Analysis

Data entry and statistical analysis were performed using the IBM^®^ Statistical Package for Social Sciences (SPSS) version 27 software (IBM Corp., Armonk, NY, USA) [42]. Descriptive analysis was used to describe the sociodemographic characteristics, clinical characteristics, and prevalence of limited HL levels of the participants. To describe the continuous data, the mean with standard deviations (SD) and median with interquartile ranges (IQR) were used for normally distributed and non-normally distributed data, respectively. Frequencies and percentages were used to describe the categorical data.

Inferential analysis was conducted to determine the factors associated with limited HL levels. Odds ratios (OR) and their 95% confidence intervals (CI) were calculated using simple logistic regression (SLogR) and multiple logistic regression (MLogR). Variables with a *p*-value of <0.05 from the SLogR were subsequently included in the MLogR. The MLogR was performed using the forward binary logistic regression method. Model fitness was checked using the Hosmer−Lemeshow goodness-of-fit test. Interactions, multicollinearity, and assumptions were also checked. Statistical significance was taken at a *p*-value of <0.05.

### 2.7. Ethical Consideration

This study was conducted in compliance with the Declaration of Helsinki and the code of ethics of the World Medical Association [43]. Ethical approval was obtained from the Research Ethics Committee of Universiti Teknologi MARA (600-TNCPI (5/1/6)) (REC/678/19) before the study commenced.

## 3. Results

### 3.1. Characteristics of Respondents

A total of 497 potential participants were approached at the academic primary care clinic. They were selected based on the inclusion and exclusion criteria. Eighty-four patients declined to participate in the study. Therefore, the final number of participants was 413. The response rate was 83.1%. Table 1 shows the sociodemographic and clinical characteristics of the respondents, stratified by their health literacy levels (limited and adequate). The median age (IQR) was 67 (8). The majority of the participants were 60–69 years old (68.3%), male (59.8%), and Malay (85.7%). Most of the respondents were married (86%), had at least a secondary school education (87.2%), and had normal ECAQ scores (97.3%). Regarding household income, 77% of the respondents were from the low-income group (B40). The median HL score and IQR was 41.7 (13.9). Out of 413 respondents, 79 (19.1% (95% CI: 15.3, 23)) had limited HL while 334 (80.9% (95% CI: 77, 84.7)) had adequate HL.

### 3.2. Factors Associated with Limited HL

Table 2 shows the results of the univariate analysis using Simple Logistic Regression. From the univariate analysis, age, marital status, education, occupation, number of children, ECAQ score, smoking status, family history of medical illness, finding medical information through the television or internet, and being accompanied to the clinic had a significant *p*-value of <0.05.

These 12 variables were included in the Multiple Logistic Regression (MLogR) model. Table 3 shows the factors associated with limited HL among elderly patients, with *p*-values < 0.05. The middle-old age (70–79 years old; Adj OR 4.05, 95% CI 2.19, 7.52) and very-old age groups (≥80 years old; Adj OR 4.36, 95% CI 1.02, 18.63) were four times more likely to have limited HL levels compared with the young-old age group. Compared with those who did not, those who found medical information on the television/internet have lower odds of having limited HL (Adj OR 0.21, 95% CI 0.05, 0.93). Furthermore, patients who had at least a secondary school education (Adj OR 0.06, 95% CI 0.02, 0.24) had a lower risk of limited HL than those with non or a primary education. The R^2^ value was 0.25, which denotes 25% of the associated factors contributed to the limited HL among our study population.

## 4. Discussion

To the best of our knowledge, this is one of the few studies conducted in a developing country to determine HL levels among elderly patients attending a primary care clinic. This study is in line with international research that recognizes the elderly as a critical population for HL research [18,20]. Furthermore, the majority of published studies on HL do not focus on elderly patients and the factors that influence them in primary care settings.

This study discovered that the prevalence of limited HL in our study population was 19.1%. This is significantly lower compared with a study among the elderly in the community of Shah Alam, Selangor, Malaysia, which reported a prevalence of 62.6% [31]. Comparable to our finding, Tiller et al. reported that the prevalence of limited HL among the German elderly in an urban setting was 27% [27]. However, studies from other countries have reported a significantly higher prevalence of limited HL among their elderly populations—Taiwan (57.6%), Germany (66.3%), and China (91.5%) [28,30,32]. Compared with all of these countries, the prevalence of limited HL was the lowest in our study. One of the reasons for this could be that our population involved patients who had chronic diseases and would have had regular access to health-care services provided by clinicians who are skilled in their field [20,44,45]. All of the doctors at this clinic were either family medicine specialists or postgraduate doctors pursuing a Master’s degree in family medicine. The ongoing clinical training and continuous professional development activities they undergo may have an effect on these patients’ HL levels [20,45,46].

We also found that in comparison with the young-old age group, the middle-old and very-old age groups were four times more likely to have limited HL. These findings are consistent with studies conducted in the United States of America, the United Kingdom, Germany, Brazil, China, Taiwan, and Vietnam, which found that older persons were more likely to have limited HL [8,22,23,24,25,26,32]. Elderly patients may process information at a slower pace and have less working memory (the ability to process multiple bits of information at the same time) [47,48]. Their reduced cognitive abilities may limit their ability to participate and comprehend the health information they receive [47,48]. In contrast, Nakayama et al.’s study in Japan found that HL increased with age [49]. They proposed that this could be due to factors involving interactions between different age populations, educational background, and active internet usage within the Japanese population [49].

Education is also an important factor for HL. From our findings, those with secondary or tertiary education levels had lower odds of having limited HL than those with lower education levels. The relationship between higher education levels and increased HL levels were also reported in recent studies among the elderly in Germany, Israel, Taiwan, and China [27,28,29,33,34,50,51]. Similar findings were reported in previous studies conducted in Malaysia among type 2 diabetes mellitus patients by Esahak et al., and among overweight housewives by Shahrir et al. [50,51]. Those with higher education levels presumably have better knowledge to empower themselves to take control of their health care and subsequently increase their HL level [50]. It is presumed that people with a higher level of education are more likely to have health-seeking behaviors and critical thinking, which drives them to obtain more health-related information and increase their HL level [50]. This is a predicted outcome because older individuals, especially in developing countries, are more likely to have limited access to education, especially in impoverished countries [17,23,28]. An example is Taiwan, where many adults over the age of 85 are illiterate and have never attended school because schooling was not made compulsory to them [28]. However, several studies observed that there is no link between education level and HL level [48,49]. Nielsen-Bohlman and colleagues suggested that even persons with high literacy skills may struggle to understand health information if they lack the knowledge to understand the medical jargon used and the context of the interactions with healthcare practitioners [2].

In this digital age, technology has become one of the most important components to empower people to take control of their health. Consistent with this, our study found a significant association between lower risks of limited HL and finding medical knowledge via the television and internet. Therefore, the elderly can enhance their HL and be motivated to learn more by using the internet or television. Television, according to Li et al., can be used to assist individuals to learn more about their health [29]. Those who received health information through television had a better probability of improving their HL [29]. Van Hoa et al. also found that elderly people in Vietnam who used the internet frequently had a higher level of HL [23]. The internet is a valuable resource to find information. According to a study by Levy et al., lack of internet usage is a factor that contributed to having a limited HL level [35]. In Malaysia, Shahrir et al. found that lack of internet connectivity was linked to having limited HL levels [51]. The accessibility to health information via the mass media and internet is linked to health behavior, information seeking, and ultimately health outcomes of patients [52,53,54]. This health information accessibility has become one of the most important factors impacting the HL level. The digital platform provides people with health information to self-evaluate and make decisions about their health care, disease prevention, and health promotion [52,54]. However, the general public may need guidance from health practitioners or authorities regarding reputable sources of validated medical information.

### 4.1. Strengths and Limitations

To the best of our knowledge, this is the first study to determine the prevalence of limited HL and its associated factors among elderly patients in a primary care setting in Malaysia. However, this study has several limitations. First, because of the cross-sectional study design, the study findings could only show association but not causality. Other than that, this study was conducted in an urban university primary care setting, where the majority of participants were of Malay ethnicity, and all doctors were either family medicine specialists or postgraduate doctors pursuing a Master’s degree in family medicine. Therefore, the findings may not be generalizable to all primary care settings in Malaysia. Finally, several other factors including depression symptoms and health impairments involving vision, hearing, life-space mobility, and physical performance, which may affect HL levels, were not included in this study. Thus, the results from the multiple logistic regression should be interpreted based on the variables included in the regression model.

### 4.2. Implications on Clinical Practice and Future Research

Our study findings have several implications on clinical practice. Firstly, clinics utilizing an electronic medical records (EMR) system can program their system to discreetly “flag” elderly patients aged ≥70 years old, or those with no formal or a low education level. These cues will alert doctors of the possibility that these patients may have limited HL. Doctors can then consciously use communication strategies that may increase these patients’ understanding about their medical condition and improve their health literacy. These strategies can be divided into verbal communication, visual aids, and patient self-management and empowerment. Verbal communication strategies include using plain language, limiting information amount and assessing patients’ comprehension [55,56]. Using visual aids such as simple pictures or videos may also improve patients’ understanding of their condition or a medical procedure [55]. Examples of patient self-management and empowerment strategies include encouraging patients to ask two to three questions during their appointments, and assessing patients’ understanding of their medications [55].

Secondly, doctors should encourage elderly patients to seek medical information via mass media or the internet. These patients should be guided to reputable sources and websites to get medical information, such as the Ministry of Health Malaysia’s MyHealth website. However, to produce effective and easily accessible sources of medical information, collaboration involving the health and information technology, communication, and education sectors need to be strengthened. These contents need to be written at an appropriate reading level (generally 10–11 years old) according to The National Work Group on Literacy and Health [56], using short sentences with minimal medical jargon, and using bulleted lists instead of blocks of text [55]. These contents should also be formatted to suit smartphone interfaces, as most Malaysians access the internet through their smartphones [57].

To facilitate the elderly and the wider population to access health-related information, the Malaysian government should continue to adopt policies to improve the affordability of gadgets to access the internet. In 2013, as part of the National Broadband Initiative (NBI), a one-off rebate of RM200 was given for the purchase of a 3G smartphone, which benefited around 1.5 million youths of Malaysia [58]. Similar initiatives should be offered to the elderly population. According to the latest statistics from the Malaysian Communications and Multimedia Commission (MCMC), mobile broadband subscriptions were ten times higher compared with fixed-broadband subscriptions in Malaysia [59]. However, the Hand Phone Users Survey 2018 reported that network coverage and internet speed were still the most highly cited reasons for mobile broadband data user dissatisfaction [60]. Network problems were the main reason for complaints regarding high-speed broadband services [61]. Hence, mobile and fixed broadband internet infrastructures should continue to be improved in order to increase their coverage for the entire country, including rural areas. Reassuringly, actions are underway that are in line with the National Broadband Initiative and National Fiberisation and Connectivity Plan (NFCP) 2019–2023 [58,62]. The NFCP aims to provide affordable and high levels of high quality broadband internet connectivity to the entire country by 2023 [62].

The Ministry of Health should consider establishing policies that promote health literacy in written, multimedia, and internet-based communication directed to the public. According to the World Health Organization’s publication titled “Health Literacy: The Solid Facts”, the literacy demands of health systems and the literacy skills of average adults are mismatched [63]. To address this, several strategies that can be applied in health care facilities include simplifying the written language used for signages, forms, patient information leaflets, medication instructions, and discharge instructions [63].

Regarding implications for future research, our study findings on the factors that influence older people’s HL has created more areas for in-depth research. Future research involving other primary care clinics in Malaysia that include other ethnic groups and rural populations should be performed to confirm the findings of this study. Other factors that may be associated with HL level, including depressive symptoms and health impairments involving vision, hearing, life-space mobility, and physical performance, should be studied. A study assessing HL-related knowledge, attitude, practices, and perceived barriers among primary care doctors in Malaysia is currently underway in order to better understand the other side of the HL interface.

## 5. Conclusions

In conclusion, the prevalence of limited HL levels among the elderly patients in this academic primary care clinic was low. Elderly patients with a good educational background and the ability to find information through the internet or television were found to be associated with lower risks of having limited HL levels, while increased age was associated with higher odds of having limited HL. These factors should be considered in future interventions in order to improve the health of these patients.

## Figures and Tables

**Table 1:** Sociodemographic and clinical characteristics of the overall participants and according to health literacy level (*N* = 413).

Characteristics	Health Literacy Level *		Total
	Limited	Adequate	
	*N* = 79	*N* = 334	*N* = 413
	*n* (%)	*n* (%)	*n* (%) **
Health literacy level			
Limited	79 (100%)	-	79 (19.1%)
Adequate	-	344 (100%)	334 (80.9%)
Age (years): Median [IQR]			67 (8)
Age Group: *n* (%) years			
Young-Old Age (60–69)	29 (36.7%)	253 (75.7%)	282 (68.3%)
Middle-Old Age (70–79)	42 (53.2%)	75 (22.5%)	117 (28.3%)
Very-Old (≥80)	8 (10.1%)	6 (1.8%)	14 (3.4%)
Gender: *n* (%)			
Male	42 (53.2%)	205 (61.4%)	247 (59.8%)
Female	37 (46.8%)	129 (38.6%)	166 (40.2%)
Ethnicity: *n* (%)			
Malay	66 (83.5%)	288 (86.2%)	354 (85.7%)
Others	13 (16.5%)	46 (13.8%)	59 (14.3%)
Marital status: *n* (%)			
Single, widow, and divorcee	22 (27.8%)	36 (10.8%)	58 (14%)
Married	57 (72.2%)	298 (89.2%)	355 (86%)
Education level: *n* (%)			
No formal education and primary education	37 (46.8%)	16 (4.8%)	53 (12.8%)
Secondary and tertiary	42 (53.2%)	318 (95.2%)	360 (87.2%)
Household income: *n* (%)			
B40 (RM < 4850)	66 (83.5%)	252 (75.4%)	318 (77%)
M40/T20 (RM ≥ 4850)	13 (16.5%)	82 (24.6%)	95 (23%)
Occupation: *n* (%)			
Employed	8 (10.1%)	32 (9.6%)	40 (9.7%)
Retired/Pensioner	42 (53.2%)	269 (80.5%)	311 (75.3%)
Unemployed	29 (36.7%)	33 (9.9%)	62 (15.0%)
Children: *n* (%)			
No	0 (0.0%)	11 (3.3%)	11 (2.7%)
Yes	79 (100.0%)	323 (96.7%)	402 (97.3%)
Living arrangement: *n* (%)			
Stays alone	1 (1.3%)	7 (2.1%)	8 (1.9%)
Stays with family members	78 (98.7%)	327 (97.9%)	405 (98.1%)
Smoking status: *n* (%)			
Non-smoker	63 (79.7%)	223 (66.8%)	286 (69.2%)
Former smoker	14 (17.7%)	80 (24%)	94 (22.8%)
Smoker	2 (2.5%)	31 (9.3%)	33 (8%)
Alcohol consumption: *n* (%)			
No	77 (97.5%)	315 (94.3%)	392 (94.9%)
Yes	2 (6.1%)	19 (5%)	21 (5.1%)
Elderly Cognitive Assessment Questionnaire (ECAQ) score: *n* (%)			
7–10: Normal	69 (87.3%)	333 (99.7%)	402 (97.3%)
<6: Borderline/Probable	10 (12.7%)	1 (0.3%)	11(2.7%)
Medical illness: *n* (%)			
No	2 (2.5%)	20 (6%)	22 (5.3%)
Yes	77 (97.5%)	314 (94%)	391 (94.7%)
Number of medical illnesses: *n* (%) (*n* = 391)			
1	11 (14.3%)	59 (18.8%)	70 (17.9%)
2	15 (19.5%)	96 (30.6%)	111 (28.4%)
≥3	51 (66.2%)	159 (50.6%)	210 (53.7%)
Family History of Medical Illness: *n* (%)			
No	30 (38%)	72 (21.6%)	102 (24.7%)
Yes	49 (62%)	262 (78.4%)	311 (75.3%)
Family members with medical training: *n* (%)			
No	56 (70.9%)	247 (74%)	303 (73.4%)
Yes	23 (29.1%)	87 (26%)	110 (26.6%)
Perceived health status: *n* (%)			
Very poor/poor	6 (7.6%)	24 (7.2%)	30 (7.3%)
Fair	32 (40.5%)	118 (35.3%)	150 (36.3%)
Good	37 (46.8%)	160 (47.9%)	197 (47.7%)
Very Good	4 (5.1%)	32 (9.6%)	36 (8.7%)
Accompanied by family to the clinic: *n* (%)			
No	21 (26.6%)	150 (44.9%)	171 (41.4%)
Yes	58 (73.4%)	184 (55.1%)	242 (58.6%)
Finding medical information on television (TV) or the internet: *n* (%)			
No	36 (45.6%)	48 (14.4%)	84 (20.3%)
Yes	43 (54.4%)	286 (85.6%)	329 (79.7%)
Limitations on activities: *n* (%)			
No	59 (74.4%)	271 (81.1%)	330 (79.9%)
Yes	20 (25.3%)	63 (18.9%)	83 (20.1%)

* Data presented as row percentage. ** Data presented as column percentage. IQR—interquartile range.

**Table 2:** Simple logistic regressions on the factors associated with limited health literacy level among elderly patients.

Characteristics	Beta (SE)	Wald (df)	OR (95% Cl)	*p*-Value
Age Group				
Young-Old (60–69)			1	ref
Middle-Old (70–79)	1.59 (0.28)	33.29 (1)	4.89 (2.85, 8.37)	<0.001 *
Very-Old (≥80)	2.45 (0.58)	18.24 (1)	11.63 (3.77, 35.87)	<0.001 *
Gender				
Male			1	ref
Female	0.34 (0.25)	1.78 (1)	1.40 (0.85, 2.29)	0.182
Ethnicity				
Malay			1	ref
Others	0.21 (0.34)	0.38 (1)	1.23 (0.63, 2.41)	0.54
Marital status				
Single/Widow/Divorce			1	ref
Married	−1.16 (0.31)	14.33 (1)	0.31 (0.17, 0.57)	<0.001 *
Education level				
No formal/primary			1	ref
Secondary/tertiary	−2.86 (0.34)	70.36 (1)	0.06 (0.03, 0.11)	<0.001 *
Household income				
B40 (RM < 4850)			1	ref
M40/T20 (RM ≥ 4850)	−0.50 (0.33)	2.33 (1)	0.61 (0.32, 1.15)	0.127
Occupation				
Unemployed			1	ref
Employed	−1.26 (0.47)	7.15 (1)	0.28 (0.11, 0.72)	0.007 *
Retired/Pensioner	−1.73 (0.30)	32.34 (1)	0.18 (0.10, 0.32)	<0.001 *
Number of children				
1–2			1	ref
≥3	1 (0.45)	4.98 (1)	2.72 (1.13, 6.55)	0.026 *
Living arrangement				
Stays alone			1	ref
Stays with family members	0.51 (1.08)	0.23 (1)	1.67 (0.20, 13.77)	0.634
Smoking status				
Non-smoker			1	ref
Former smoker	−0.48 (0.32)	2.20 (1)	0.62 (0.33, 1.17)	0.138
Smoker	−1.48 (0.74)	3.95 (1)	0.23 (0.05, 0.98)	0.047 *
Alcohol consumption				
No			1	ref
Yes	−0.84 (0.75)	1.25 (1)	0.43 (0.10, 1.89)	0.264
Elderly Cognitive Assessment Questionnaire (ECAQ)				
Normal (7–10)			1	ref
Borderline/Probable dementia (≤6)	3.88 (1.06)	13.45 (1)	48.26 (6.08, 383.19)	<0.001*
Medical illness				
No			1	ref
Yes	0.90 (0.75)	1.42 (1)	2.45 (0.56, 10.72)	0.233
Number of medical illness				
1			1	ref
2	−0.18 (0.43)	0.17 (1)	0.84 (0.36, 1.95)	0.681
≥3	0.54 (0.37)	2.20 (1)	1.72 (0.84, 3.52)	0.138
Family History of Medical Illness				
No			1	ref
Yes	−0.80 (0.27)	8.98 (1)	0.45 (0.27, 0.76)	0.003 *
Family member with medical training				
No			1	ref
Yes	0.15 (0.28)	0.31 (1)	1.17 (0.68, 2.01)	0.58
Perceived health status				
Very poor/poor			1	ref
Fair	0.08 (0.50)	0.03 (1)	1.09 (0.41, 2.88)	0.87
Good	−0.08 (0.49)	0.03 (1)	0.93 (0.35, 2.42)	0.874
Very Good	−0.69 (0.70)	0.98 (1)	0.50 (0.13, 1.97)	0.322
Accompanied by family to the clinic				
No			1	ref
Yes	0.81 (0.28)	8.56 (1)	2.25 (1.31, 3.88)	0.003 *
Finding medical information on television (TV) or the internet				
No			1	ref
Yes	−1.61 (0.28)	34.27 (1)	0.20 (0.12, 0.34)	<0.001 *
Limitations on activities				
No			1	ref
Yes	0.38 (0.29)	1.64 (1)	1.46 (0.82, 2.60)	0.2

* Statistically significant at *p* < 0.05; Statistical test—Simple Logistic Regression; OR—odds ratio; CI—confidence interval; ref—reference group.

**Table 3:** Multiple logistic regressions on the factors associated with a limited health literacy level among elderly patients.

Characteristics	Adjusted Beta (SE)	Wald (df)	Adjusted OR (95% CI)	*p*-Value
Age Group years				
Young-Old (60–69)			1	ref
Middle-Old (70–79)	1.40 (0.32)	19.7 (1)	4.05 (2.19, 7.52)	<0.001 *
Very-Old (≥80)	1.47 (0.74)	3.94 (1)	4.36 (1.02, 18.63)	0.047 *
Finding medical information on television (TV) or the internet				
No			1	ref
Yes	−1.55 (0.75)	4.26 (1)	0.21 (0.05, 0.93)	<0.001 *
Education				
No formal/Primary education			1	ref
Secondary/Tertiary	−2.83 (0.71)	15.82 (1)	0.06 (0.02, 0.24)	<0.001 *

The model reasonably fits well (Hosmer−Lemeshow test: *p* = 0.822); model assumptions were met; no significant interactions and multicollinearity problem; model explained between 24.7% (Cox and Snell R^2^) and 39.7% (Nagelkerke R^2^) of the variance in limited HL and correctly classified 87.2% of cases; sensitivity 40.5%, specificity 98.2%. * Statistical significance at *p* < 0.05; OR—odds ratio; CI—confidence interval; df—degree of freedom; ref—reference group.

## Data Availability

Data are kept at the Department of Primary Care Medicine, Universiti Teknologi MARA, in Selangor, Malaysia. Data will be shared upon request and are subject to the applicable and relevant personal data protection laws and regulations.
